# Supplementary material for: CysB Is a Key Regulator of the Antifungal Activity of Burkholderia pyrrocinia JK-SH007
Source: Int J Mol Sci. 2023 Apr 29;24(9):8067. doi: 10.3390/ijms24098067 (PMC10179380; doi:10.3390/ijms24098067)
Supplement: Supplementary file 1 [file ijms-24-08067-s001.zip › ijms-2331469-supplementary.pdf]

Supplementary

Supplementary Figures

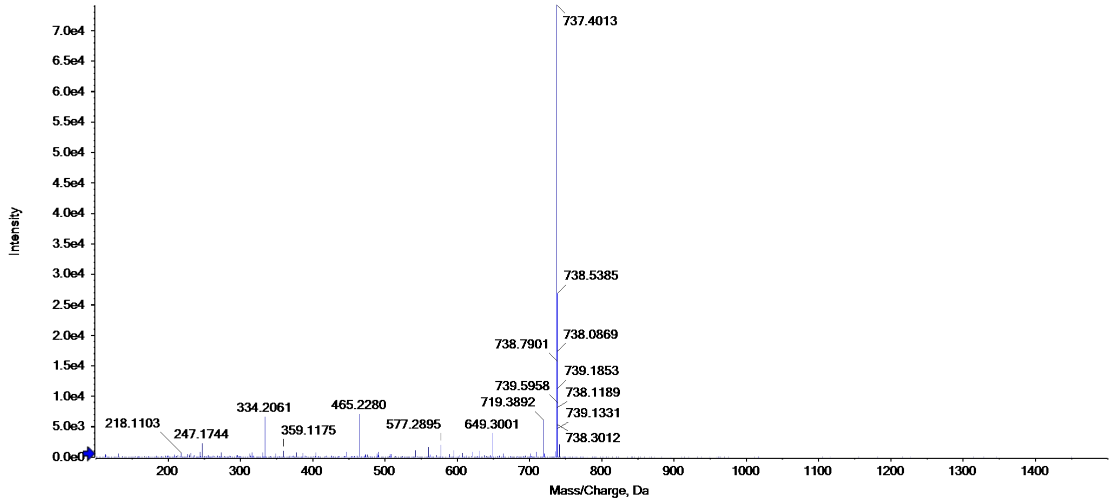

Supplementary Figure S1. Results of the MS.

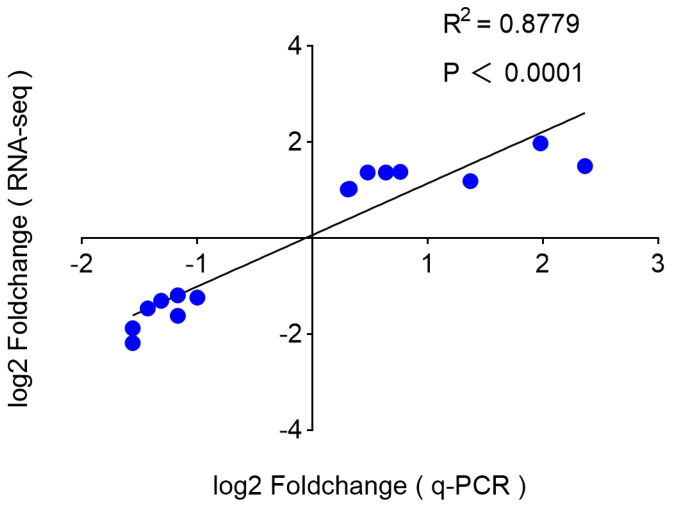

Supplementary Figure S2. Transcriptome and q-PCR correlation.

Supplementary Tables

Supplementary Table S1. Sequencing data quality preprocessing results.

Sample	raw_reads	raw_bases	clean_reads	clean_bases	valid_bases	Q30	GC
JK_007_1	16.40M	2.46G	15.92M	2.32G	94.42%	92.55%	66.49%

JK_007_2	16.44M	2.47G	15.94M	2.33G	94.66%	92.46%	67.89%
JK_007_3	16.54M	2.48G	16.01M	2.35G	94.65%	92.24%	69.56%
V49_1	16.36M	2.45G	15.68M	2.20G	89.46%	90.42%	66.62%
V49_2	16.35M	2.45G	15.70M	2.24G	91.46%	90.51%	70.54%
V49_3	16.58M	2.49G	15.89M	2.26G	90.82%	90.33%	69.32%
